# Osmotic Gradient Is a Factor That Influences the Gill Microbiota Communities in *Oryzias melastigma*

**DOI:** 10.3390/biology11101528

**Published:** 2022-10-19

**Authors:** Keng Po Lai, Delbert Almerick T. Boncan, Lu Yang, Cherry Chi Tim Leung, Jeff Cheuk Hin Ho, Xiao Lin, Ting Fung Chan, Richard Yuen Chong Kong, William Ka Fai Tse

**Affiliations:** 1 Key Laboratory of Environmental Pollution and Integrative Omics, Education Department of Guangxi Zhuang Autonomous Region, Guilin Medical University, Huan Cheng North 2nd Road 109, Guilin 541004, China; 2 Department of Chemistry, City University of Hong Kong, Hong Kong SAR, China; 3 State Key Laboratory of Marine Pollution, City University of Hong Kong, Hong Kong SAR, China; 4 State Key Laboratory of Agrobiotechnology, School of Life Sciences, The Chinese University of Hong Kong, Hong Kong SAR, China; 5 Department of Psychiatry, Icahn School of Medicine at Mount Sinai, New York, NY 10029, USA; 6 Research Centre for the Oceans and Human Health, City University of Hong Kong Shenzhen Research Institute, Shenzhen 518057, China; 7 Laboratory of Developmental Disorders and Toxicology, Center for Promotion of International Education and Research, Faculty of Agriculture, Kyushu University, Fukuoka 819-0395, Japan

**Keywords:** osmotic stress, fish gill, 16S rRNA metagenomic sequencing, stress

## Abstract

**Simple Summary:**

This study was applied to the laboratory medaka to understand how the osmotic gradient could influence the composition of the gill microbiota communities. The data suggested that the shift of the gill microbiota community has relied on the first sense of osmolality differences, and such changes were accomplished by the enriched osmosensing and metabolic pathways.

**Abstract:**

The fish gill is the first tissue that is exposed to the external media and undergoes continuous osmotic challenges. Recently, our group published an article entitled “Integrated Omics Approaches Revealed the Osmotic Stress-Responsive Genes and Microbiota in Gill of Marine Medaka” in the journal mSystems (e0004722, 2022), and suggested the possible host-bacterium interaction in the fish gill during osmotic stress. The previous study was performed by the progressive fresh water transfer (i.e., seawater to fresh water transfer via 50% seawater (FW)). Our group hypothesized that osmotic gradient could be a factor that determines the microbiota communities in the gill. The current 16S rRNA metagenomic sequencing study found that the direct transfer (i.e., seawater to fresh water (FWd)) could result in different gill microbiota communities in the same fresh water endpoints. *Pseduomonas* was the dominant bacteria (more than 55%) in the FWd gill. The Kyoto Encyclopedia of Genes and Genomes and MetaCyc analysis further suggested that the FWd group had enhanced osmosensing pathways, such as the ATP-binding cassette transporters, taurine degradation, and energy-related tricarboxylic acid metabolism compared to the FW group.

## 1. Background

Ocean freshening has been observed in the surface and intermediate waters of the open ocean [1,2]. Salinity change in the ocean could bring direct impact to marine organisms, which further highlights the possible changes in the living habitat of fishes in the future. Aquatic organisms, such as the medaka species, have developed an effective osmoregulatory mechanism to compensate for the water and ion gain or loss in various salinity environments [3,4]. On the other hand, single-celled bacteria must adjust their cellular functions and physiology to acclimate to the wide range of environmental conditions. The osmoregulatory mechanism in bacteria depends on their modifications of the extracellular barrier, transporters in the membrane, and enzyme biosynthesis [5,6]. The adaptive microbial community shifts concomitant with the host habitat change may help the host survive [7]. It is shown that bacteria in plants could assist their salinity tolerances [8]. We believe that the microbiota in the fish gill could play a similar role. Fishes contact microorganisms throughout their lifetime with beneficial or pathogenic relationships. Bacteria can be found in the external tissues such as skin and gill, and the internal organs such as the gut. Studies in gut microbes have identified the host-microbial relationship that contributes to metabolic homeostasis and immune response [9,10]. In contrast, studies in gill microbes are mainly restricted to the pathological infection events in economically valuable aquaculture fishes [11,12]. Numerous factors have been found to influence microbiota composition, such as diet composition, living habitat, and osmotic stress [3,13,14,15,16]. 

The gill is the first tissue to sense and respond to osmolality changes [17]. Gill bacteria continuously face osmotic challenges, and their osmoregulatory mechanisms are essential for survival. Our previous study has confirmed the shift of gill microbiota communities after the progressive seawater to fresh water transfer (transfer from seawater was firstly acclimated in 50% seawater for seven days, and then transferred to fresh water for seven days) [18]. Based on the result, we initiated the current study to understand how salinity could influence the microbiota communities in the fish gill. Since different bacteria strains have different salinity tolerance abilities [19], it is expected that the higher salinity differences at the first exposure could reduce the populations of those low salinity tolerant bacteria. In this follow-up study, we would like to determine the major factor that could affect the gill microbiota community during osmotic stress. One of the advantages of using the medaka model was the controlled raising conditions in the laboratory environment, such as water and feed, that could be factors affecting the result. This study applied all the same conditions, and the same batch of fish, as our previous report, which could help us to eliminate the extra environmental factors [18]. We performed a direct transfer experiment (transfer from seawater to fresh water directly, FWd) to generate a larger osmotic gradient difference. We hypothesize the difference in osmotic gradient at the first sensation could lead to various gill microbiota compositions at the same endpoint condition (fresh water). The current study would like to identify the differences in: (i) gill microbiota compositions after the progressive or direct fresh water transfer; and (ii) enriched biological functions and pathways that are contributed by the gill microbiota. 

## 2. Material and Method

### 2.1. Fish Maintenance and Experimental Setup 

Six-month-old marine medaka (*Oryzias melastigma*) with a weight between 0.35 and 0.50 g were kept in seawater at 26 °C. The transfer experiment setting was based on our previous studies [18]. In brief, fish were kept in a 20 L tank for at least a week before the transfer experiment. Thirty fish were transferred to fresh water directly for fourteen days (FWd), while seawater to seawater transfer was performed in another thirty fish as the control group (SW). Fish were kept in one tank for each condition after the transfer. The experimental duration was 2 weeks. Gill samples were extracted afterwards. Five gill samples were pooled as one sample, and each group contained four replicates for 16S rRNA metagenomics sequencing. The experimental protocols were approved by the ethics committee of Kyushu University, Japan (A19-165-1). 

### 2.2. 16S rRNA Metagenomics Sequencing

The gill samples were digested with the ATL lysis buffer with proteinase K from the DNeasy Blood and Tissue Kit Mini Kit (Qiagen, Hilden, Germany) at room temperature for bacterial DNA extraction. The DNA was recovered by the AE elution buffer and stored at −20 °C. Rearing water was collected for extraction as well. Bacterial DNA was then quantified by the Qubit dsDNA HS Assay Kit (Life Technologies, Carlsbad, CA, USA) as previously described [18]. Briefly, a 30 ng genomic DNA sample was used for amplicon polymerase chain reaction (PCR) reaction. The V3 and V4 regions of the 16S rRNA gene were amplified by the following PCR setting: 95 °C for 3 min as initial denaturation; followed by 25 cycles of 30 s denaturation (95 °C), 30 s annealing (55 °C), and 30 s elongation (72 °C); and ending with the final elongation step at 72 °C for 5 min. The sequences of the primers were: forward, 5′-TCG TCG GCA GCG TCA GAT GTG TAT AAG AGA CAG CCT ACG GGN GGC WGC AG-3′; reverse 5′-GTC TCG TGG GCT CGG AGA TGT GTA TAA GAG ACA GGA CTA CHV GGG TAT CTA ATC C-3′. The 16S V3 and V4 amplicon was purified from free primers and primer-dimer species using Ampure XP beads (Agencourt Bioscience, Beverly, MA, USA). The quality of the library was checked by the Agilent 2100 bioanalyzer (EvaGreen, Santa Clara, CA, USA) before sequencing. The BGI sequencer platform was used in this study. The sequencing data are available in the NCBI BioProject PRJNA702883.

### 2.3. Bioinformatics Analysis, Data Processing, and ASV Prediction

All the downstream analysis settings were the same as in our previous study [18]. In brief, custom-made Python, Perl, and R scripts were used in the bioinformatics analysis. Adapters, primers, and low-quality bases were trimmed by using either a standalone or combination of FastQC (v0.11.8, Babraham Bioinformatics, Cambridge, UK) Cutadapt v2.10, and Trim Galore v0.6.6. The minimum Phred quality score is 20 for 150 bp paired end (PE) reads. Qiime v2 (Qiime2; 2020.6 version) was used for the taxonomy classification. The RESCRIPt program in Qiime 2 was applied. It was based on the 341F-805R universal primer in the Silva 138.1 SSU NR99 reference database (Silva 138) [20]. A quality control step was accomplished by Qiime2 ‘quality-control exclude seqs’ module that set a 97% identity threshold and 95% query alignment with vsearch. ASVs in Qiime 2 were used for phylogenetic analysis. 

### 2.4. Taxonomic and Functional Analysis 

The Phyloseq package in R 4.0.2 was used for taxonomic profiling [21]. A significant difference on alpha diversity analysis was performed by one-way ANOVA with post-hoc Tukey HSD at 95% confidence level. DESeq2 was used to identify the significant differences in abundant taxa. Functional profiling was performed by EC/MetaCyc and KEGG/KO databases in PICRUSt2 v2.0.0-b, while MetaCyc and KEGG pathway analyses were performed by STAMP v2.1.3 [22]. Statistical significance in pathway enrichment was based on White’s non-parametric t-test (two-sided) and Storey’s FDR for multiple testing. Co-occurrence network analysis at the genus level was analyzed by Calypso v8.84 with default parameters [23].

## 3. Results and Discussion

**Figure 1:**
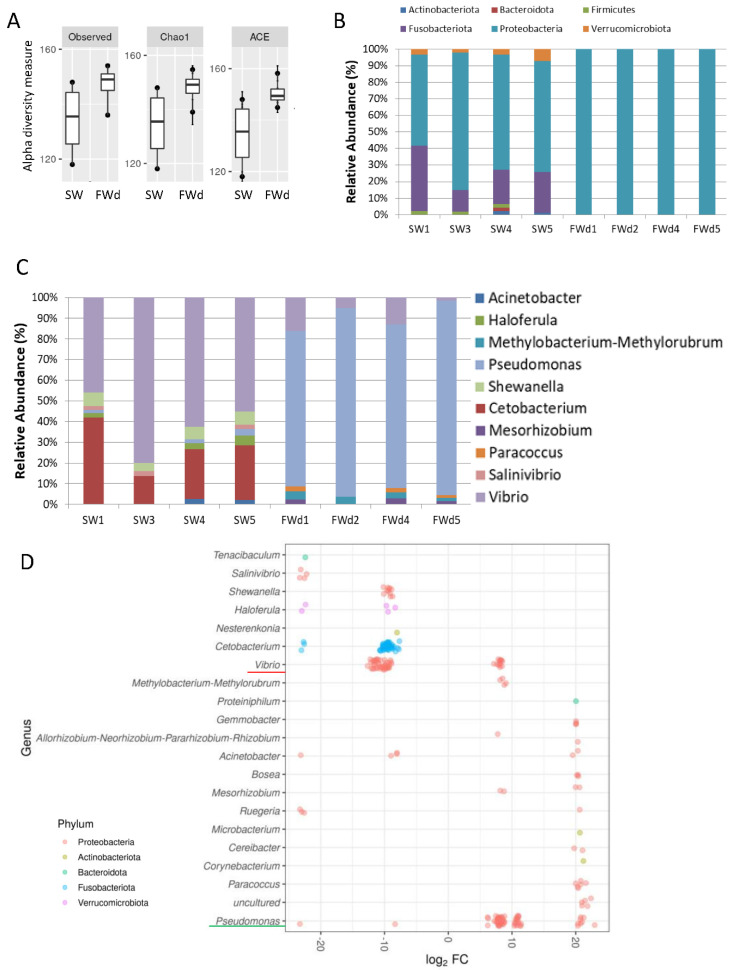
**Changes in gill microbiota composition after direct fresh water transfer**. (**A**) Various alpha diversity measurements between the SW and FWd groups. Results indicated that the direct fresh water transfer did not cause any significant changes of gill microbial diversity. (**B**) Microbiota communities at phylum level in SW and FWd gills. *Proteobacteria* were the dominant bacteria (>99%) in FWd. (**C**) Microbiota communities at genus level in SW and FWd gills. The major bacteria changed from *Vibrio* to *Pseudomonas* after the direct fresh water transfer. (**D**) Volcano plot showing a total of 21 bacteria at genus level were changed after the direct fresh water transfer. Decrease in *Vibrio* and *Cetobacterium* and increase in *Pseudomonas* were spotted. *Y*-axis shows the genus, and the *X*-axis represents the log_2_ fold value.

**Figure 2:**
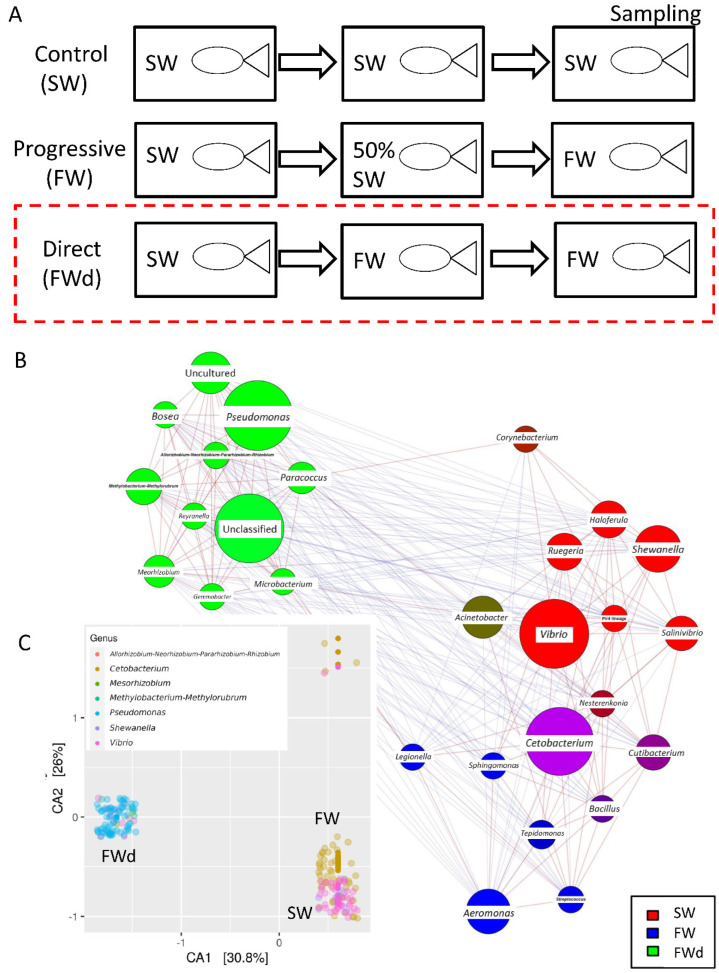
**Composition of microbiota communities in the three conditions.** (**A**) Experiment setting of the study. The red box indicates the current new data of direct fresh water transfer (FWd). The data analysis among the three conditions was based on our previous reported data (SW, and FW). (**B**) The network relationship among the three groups was obtained from Calypso analysis (SW in red, FW in blue, FWd in green). (**C**) Distinct microbiota diversity patterns among the SW (*Vibrio*, pink spots at right bottom corner), FW (*Ceteobacterium*, yellow-orange at the right), and FWd (*Pseudomonas*, blue at the left).

**Figure 3:**
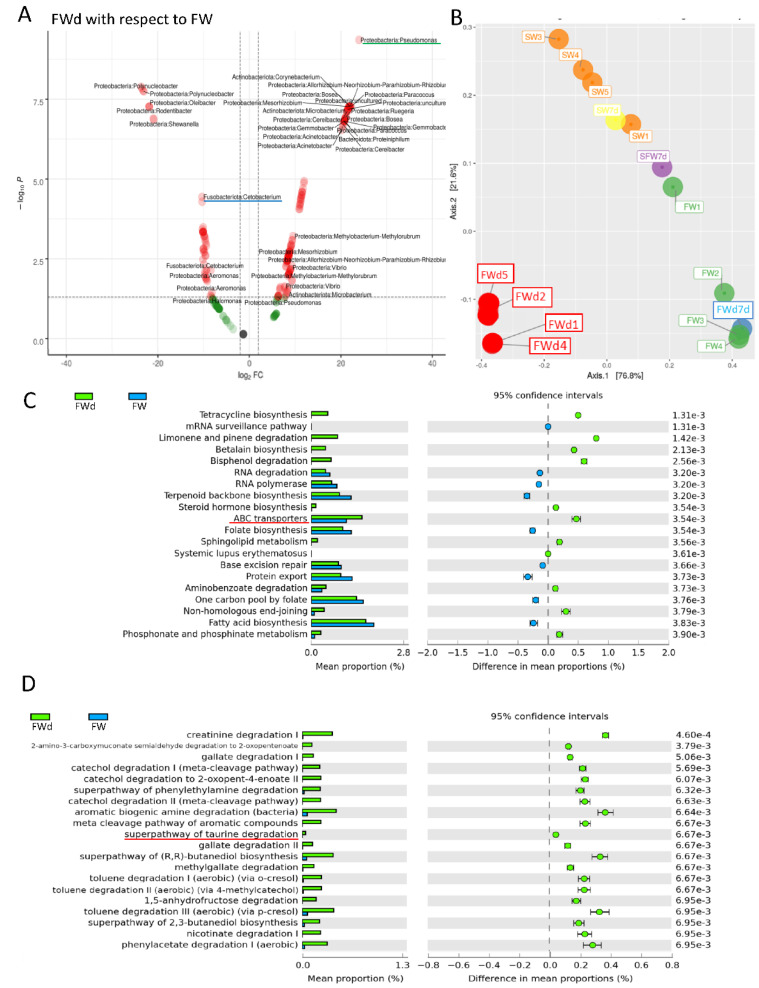
**Differences of gill microbiota between FW and FWd groups.** (**A**) Comparison of microbiota at genus level between FW and FWd. Volcano plot of the microbiota (FWd with respect to FW). Red indicates the significant changes in abundances, while green refers to changes without statistical significance. The FWd gill had a higher relative abundance (right) of *Pseudomonas* (red underlined), but lower abundance (left) of *Cetobacterium* (green underlined). (**B**) PCoA analysis of rearing water and gill samples. The gill microbiota were located apart from the rearing water (modified from our published work [18]), newly added FWd gill samples (red), and FWd water samples (blue). (**C**) KEGG bioinformatics analysis identified various enriched pathways between FWd and FW groups. The top 20 enriched pathways are shown. Osmosensing related ABC transporters were enriched in FWd group. The full list can be referred to in Supplementary File S4. (**D**) MetaCyc analysis revealed various enriched degradation pathways in Fwd group, such as creatinine degradation I and taurine degradations. Furthermore, the TCA cycle was enriched, which can be referred to in Supplementary File S5.

The 16S rRNA metagenomics sequencing analysis on gill identified a total of 495 sequences (244 in SW, and 251 in FWd) by the amplicon sequence variant (ASV) method. The complexity of species diversity was analyzed by the Alpha diversity [24], and no significant differences were spotted in the tested parameters (Observed, Chao1, and ACE) (Figure 1A). Such a result was different from our previous study that identified significant reduced diversity after progressive transfer [18]. In addition, changes in the gill microbiota were found. At Phylum level, the dominant bacteria in the control marine medaka (SW) were *Proteobacteria* and *Fusobacteriota*. After the direct fresh water transfer (FWd) experiment, *Fusobacteriota* were eliminated and the whole community was monopolized by *Proteobacteria* (over 99% among the identified microbiota) (Figure 1B). At genus level, *Vibrio* (~55%) and *Cetobacterium* (~24%), were the major microbiota in SW, and the dominant bacteria shifted from *Vibrio* (~5% in FWd) to *Pseudomonas* (from less than 2% in SW to about 57% in FWd) in the FWd. Moreover, *Cetobacterium* disappeared after the direct transfer (Figure 1C). The full list of the microbiota with abundance can be referred to in the Supplementary File S1. To further visualize such changes, a volcano plot showed a total of 21 bacteria at genus level were changed after the direct fresh water transfer (Figure 1D). Further bioinformatics analyses (KEGG and MetaCyc analysis) were performed, and numerous metabolic pathways were enriched, such as the steroid hormone biosynthesis and TCA cycle (Supplementary File S2 for KEGG and Supplementary File S3 for MetaCyc). Regarding the composition, we presented totally different gill microbiota communities in this direct fresh water transferred dataset (FWd), when compared to the progressive fresh water transfer (FW, Figure 2A). Calypso analysis was performed to obtain the network relation among the three groups. The progressive transfer group (FW, blue) shared some similarity (purple) with the control group (SW, blue), while the direct transfer group (FWd, green color) stands alone (Figure 2B). Furthermore, the canonical correspondence analysis (CCA) plot displayed distinct distributions among the SW (right bottom corner), FW (middle right), and FWd (left bottom corner) groups, in which the majority bacteria were *Vibro* in SW, *Ceteobacterium* in SFW, and *Pseudomonas* in FWd. The result also demonstrated a closer relationship between SW and FW groups (Figure 2C). Regarding the microbiota compositions, the hypotonic stress (either direct or progressive transfer) could result in a decrease in *Vibrio* and an increase in *Pseudomonas* in the gill. However, when we further compared the two populations in FWd and FW, the FWd group had a significantly higher abundance of *Pseudomonas* and *Vibrio* than FW (Figure 3A). *Vibrio* were known to be the most dominant microbiota in marine fish, while *Pseudomonas* were mainly found in fishes living in fresh water [3]. This study identified that *Pseudomonas* contributed around 57% of the microbiota in the FWd group. This bacteria is known for producing digestive enzymes such as protease and lipase [25]. On the other hand, *Cetobacterium* was eliminated in the FWd group, which was different from our previous progressive transfer (FW) data. The result suggested that *Cetobacterium* might not be able to switch on their osmoregulatory mechanism for survival upon the drastic salinity changes in the surrounding environment. Moreover, to understand the origins of those FWd gill bacteria, 16S rRNA metagenomics sequencing was performed in the fresh water sample. The PCoA result showed no overlapping between the microbiota in external fresh water media (blue) and the FWd gill samples (red) (Figure 3B). Such a result further confirmed that the change in external aquatic microbiota composition was not the major factor that contributed the shift of gill bacteria as reported [18]. 

Bacteria sense the change of external osmolality and response via regulating water fluxes across the cytoplasmic membrane [26]. One of the major strategies for microbes to survive through the osmotic challenges is the regulation of intracellular solute concentrations [27]. Osmosensing transporters in bacteria could sense the changes in extracellular osmotic pressure and thus modify the uptake of organic osmolytes [28]. In this report, we further compare the functional differences of microbiota that presented in the progressive transfer and direct transfer groups. In the KEGG analysis, ATP-binding cassette (ABC) transporter was enriched in the FWd group (Figure 3C). The ABC transporter (OpuA) is a response to water stress that acts as a sensing mechanism [29]. Osmotic stress affects different components in the plasma membrane in bacteria, which induces the corresponding sensors to activate the downstream osmoregulatory mechanism [30]. The full list of the enriched KEGG pathways is shown in Supplementary File S4. In addition, metabolites have been suggested to play important roles in osmoregulation. The MetaCyc analysis identified various osmo-responsive pathways, such as creatinine and taurine degradation pathways, in the FWd group. Activation of immune response in fresh water transferred eel gill has been reported [31]. Creatinine is metabolized from creatine [32], and it has immunosuppressive properties [33]. It could down-regulate the pro-inflammatory cytokine, such as tumor necrosis factor-alpha, in macrophage. The enriched degradation pathway may strengthen the immune system for acute fresh water acclimation. Although a metabolomics study in tongue sole gills demonstrated the change of creatine upon osmotic stress [34], there is limited evidence showing the direct relationship between the creatinine and osmotic stress. Further studies have to be performed to confirm such an event. Nevertheless, the bioinformatics analysis also identified several osmotic-related pathways such as taurine and myo-inositol. Taurine is one of the most common organic osmolytes, and its relative sodium-chloride-taurine transporter was found to be upregulated upon hypertonic stress in eel gill cell culture [35]. The enriched taurine degradation pathway in the FWd gill suggested that the hypertonic responsive osmolytes might not be required in rapid hypotonic challenges in the microbiota. In addition, osmotic stress in tilapia gill could influence the mRNA level of myo-inositol phosphate synthase that participates in myo-inositol metabolism [36]. Another study in tilapia larvae suggested that the myo-inositol biosynthesis pathway is critical for compensating the hyperosmotic stress [37]. Our hypo-osmotic data indicated the enrichment of the myo-inositol deregulation pathway, which matches with the pervious findings. Lastly, the comparison further spotted the enrichment of the tricarboxylic acid (TCA) cycle in the FWd group (full list is shown in Supplementary File S5). The TCA cycle is a series of chemical reactions to generate various energy-containing compounds via inter-conversion of various biomolecules during cellular respiration. The TCA cycle was found to be induced in olive flounder gill after different environmental stress, such as cadmium exposure [38], parasite infection [39], and hypoxia [40]. A study in *Corynebacterium* *glutamicum* demonstrated the increase of ATP maintenance coefficients with osmolality [41]. It supported the idea that higher energy is needed to maintain the cellular function under osmotic stress [42].The TCA cycle was enriched significantly in FWd when compared to FW, indicating the first sense of osmolality differences could influence the energy metabolism for gill microbiota acclimation. 

## 4. Conclusions

The result suggested that the recomposing of gill microbiota upon osmotic stress likely relies on the first sensation of the osmolality differences. Moreover, the direct transfer from seawater to fresh water could have shaped a brand-new microbiota community, in which more enriched pathways were identified than in progressive transfer. Further investigation should be performed to unfold the gill-bacteria relationship in osmotic stress. 

## Data Availability

The sequencing data from this study have been submitted to the NCBI bioproject (https://www.ncbi.nlm.nih.gov/bioproject; accessed on 13 September 2022) under the accession number PRJNA702883.

## Supplementary Materials

The following supporting information can be downloaded at https://www.mdpi.com/article/10.3390/biology11101528/s1, **Supplementary File S1**: Relative abundances of gill microbiota at each level. **Supplementary File S2**: KEGG analysis between FWd/SW. **Supplementary File S3**: MetaCyc analysis between FWd/SW. **Supplementary File S4**: KEGG analysis between FWd/FW. **Supplementary File S5**: MetaCyc analysis between FWd/FW.
