# Supplementary figures and images for: Osmotic Gradient Is a Factor That Influences the Gill Microbiota Communities in Oryzias melastigma
Source: Biology (Basel). 2022 Oct 19;11(10):1528. doi: 10.3390/biology11101528 (PMC9598346; doi:10.3390/biology11101528)

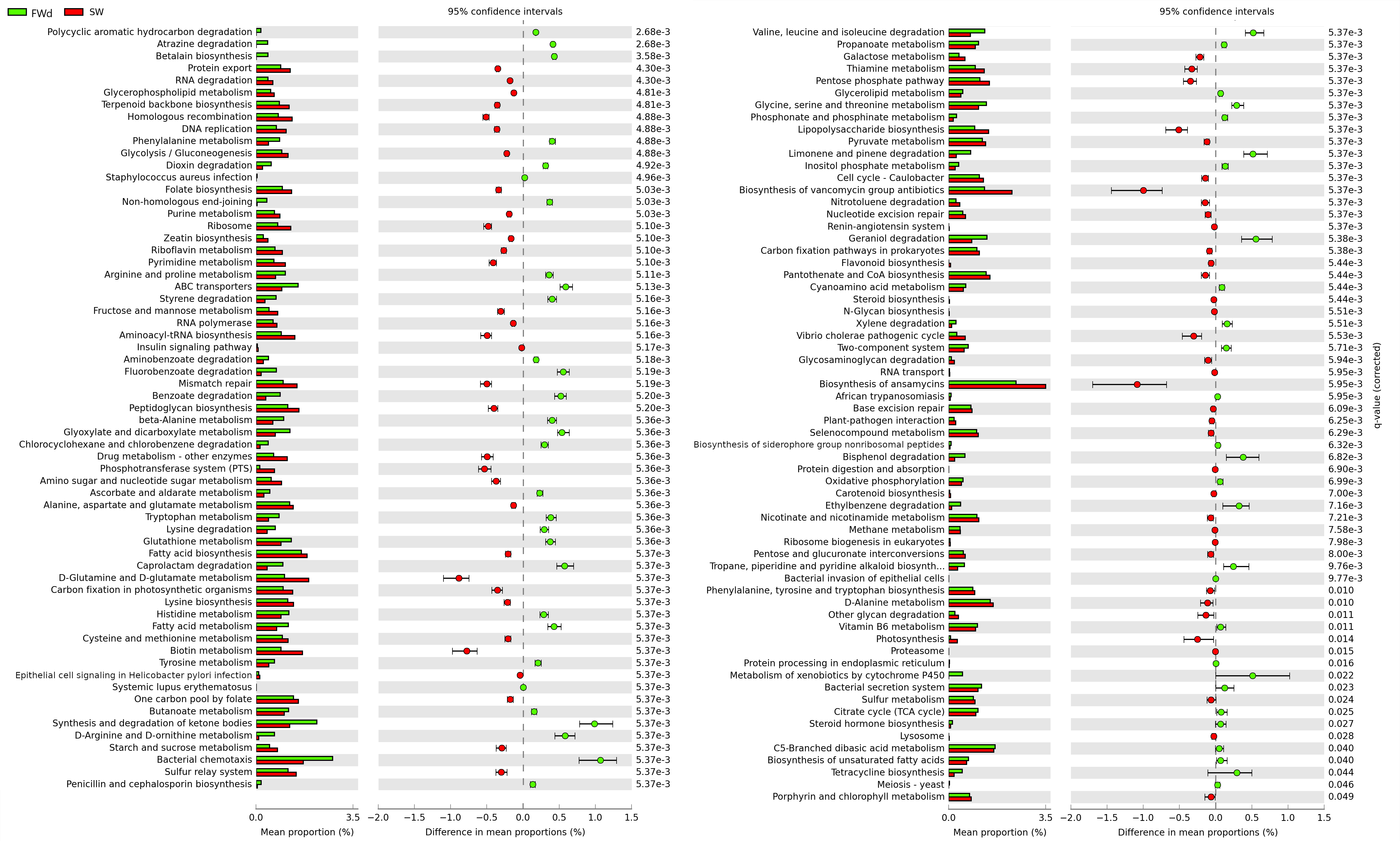

Supplement: Supplementary file 1 [file biology-11-01528-s001.zip › S2. KEGG analysis between FWd and SW.tif]

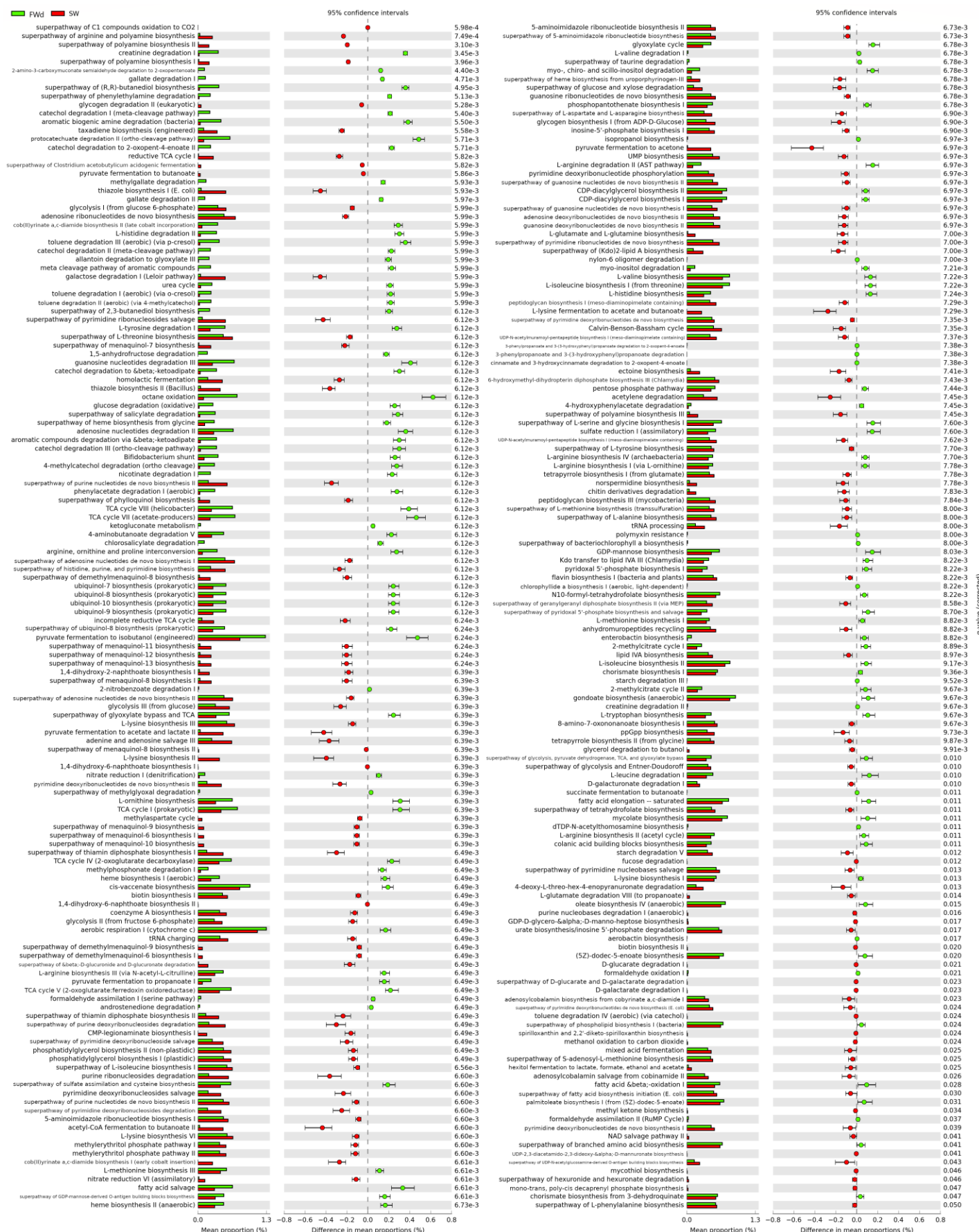

Supplement: Supplementary file 1 [file biology-11-01528-s001.zip › S3. MetaCyc analysis between FWd and SW.pdf]

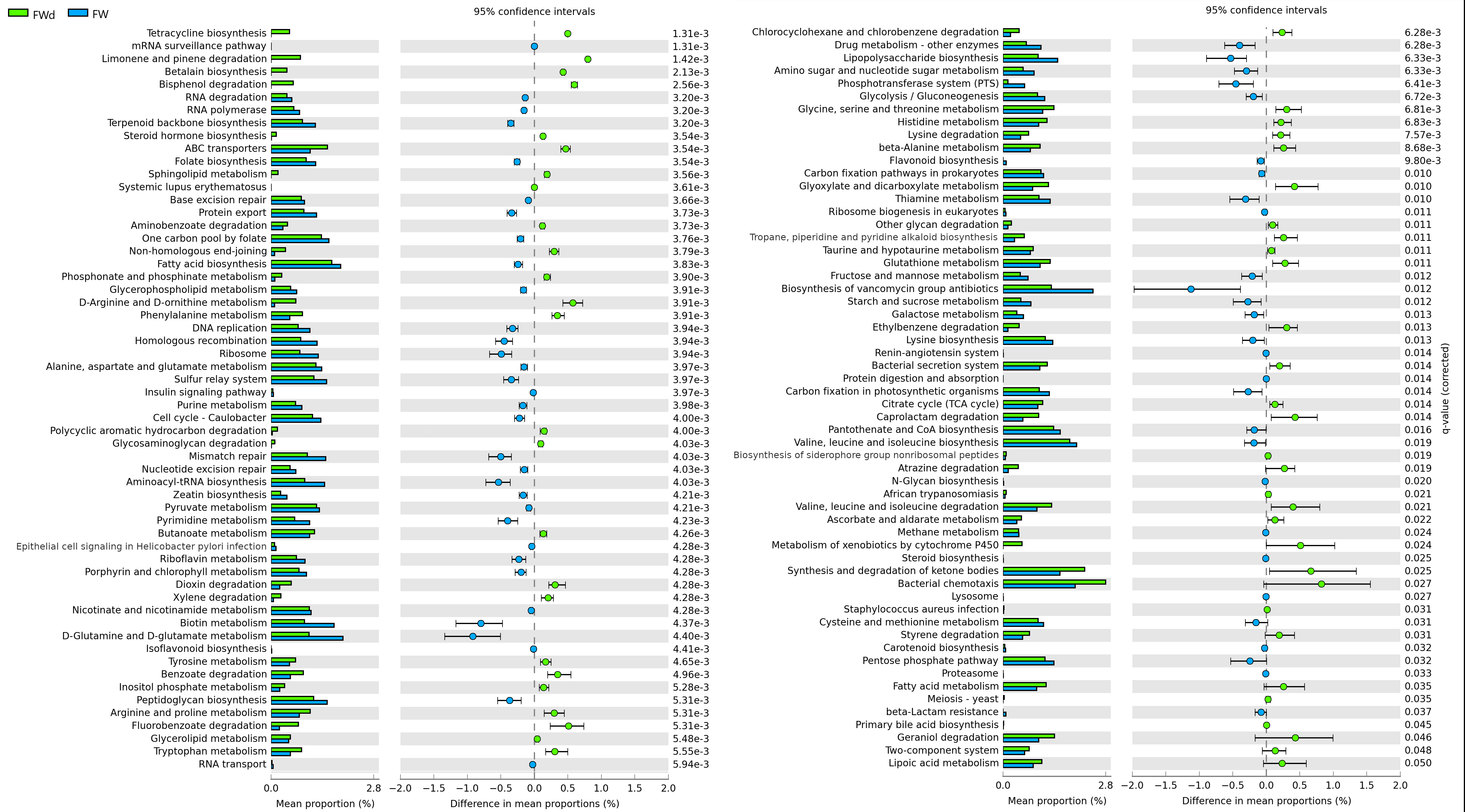

Supplement: Supplementary file 1 [file biology-11-01528-s001.zip › S4. KEGG analysis between FWd and FW.tif]

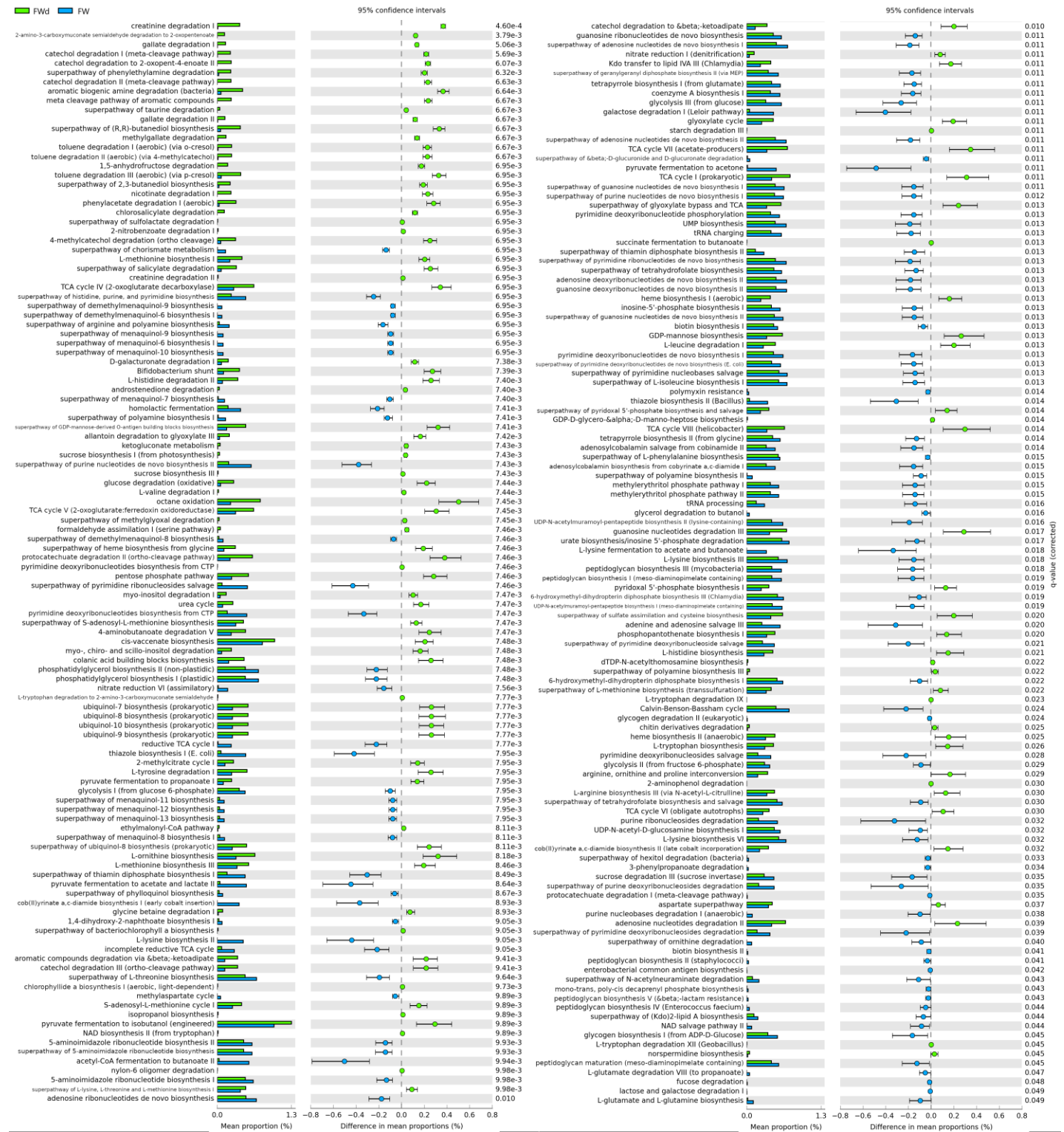

Supplement: Supplementary file 1 [file biology-11-01528-s001.zip › S5. MetaCyc analysis between FWd and FW.pdf]
